# Improving nurses’ performance in the safe handling of antineoplastic agents: a quasi-experimental study

**DOI:** 10.1186/s12912-021-00771-4

**Published:** 2021-12-09

**Authors:** A Nouri, M Seyed Javadi, E Iranijam, M Aghamohammadi

**Affiliations:** 1 Department of Nursing, School of Nursing and Midwifery, Ardabil University of Medical Sciences, Ardabil, Iran; 2 Department of Nursing, Moghan School of Nursing and Midwifery, Ardabil University of Medical Sciences, Moghan, Iran; 3 Department of Internal Medicine, School of Medicine, Ardabil University of Medical Sciences, Ardabil, Iran

**Keywords:** Antineoplastic drugs, Knowledge, Oncology nurse, Performance, Standard guidelines

## Abstract

**Background:**

The safe and standard handling of antineoplastic drugs can reduce the effects of occupational exposure and promote safe behaviors in nurses. Thus, the present study aimed to determine the effects ofstandard guidelines education on the safe handling of antineoplastic drugs among oncologynurses in Ardabil, Iran.

**Methods:**

Thequasi-experimental study with a one-group pretest-posttest design was performed among 32 nursesworking in the oncology wards of two educational hospitals in Ardabil city, during 2020. Allthe nurses in the wards who met the inclusion criteria participated in the study. The data were collectedby usinga demographic information form and nurses’knowledge assessment questionnaire regardingthe standard guidelines for working with antineoplastic drugs, and a standard checklist for examiningtheir performance in this regard. Subsequently, they were analyzed by descriptive (mean and standard deviation) and inferential statistics (t-test)and Pearson’s correlation coefficient) in SPSS 22.

**Results:**

The mean and standard deviation of the knowledge and performance scores of the oncology nurses was59.56±6.41and 18.96±2.54 respectively, which changed to 66±4.82 and 32.03±2.45 respectively three months after training. The results of the t-test represented a statistically significant difference between the level of knowledge and performance before and after the intervention (*P*=0.001).

**Conclusions:**

Based on the results, the standard guidelines education improved the nurses’ knowledge and performance on the safe handling of antineoplastic drugs in the chemotherapy wards. Therefore, it is advised to increase the awareness of the oncology nurses in this regard in the planning and policy-making ofhealthcare centers.

## Introduction

Cancer is the leading cause of death worldwide, accounting for an estimated 10 million deaths, in 2020 [1]. According to the latest reports of Iran’s Ministry of Health, cancer is currently the third leading cause of death in Iran after coronary heart disease and traffic accidents, which is becoming the second causebecause of aging of the population and decreasing deaths due to accidents [2]. Antineoplastic drugs are cytotoxic agentswhich are widely used in healthcare centers to treat patients with cancer [3].Researchers have confirmed that theadverse drug reactions (ADRs) associated with antineoplastic agents may occur in both patients and individuals involved in the treatment chain [4, 5]. The US National Institute of Occupational Safety and Health (NIOSH) classified these drugs as high risk since they are mutagenic, teratogenic, and carcinogenic [6].

Nurses are at risk for the harmful effects of antineoplastic drugs due to long-term occupational exposure [7]. They play the most important role in providing carefor cancer patients, and have the followingroles: safe treatment andmanagement of ADRs, training patients and their families on the potential ADRs, and providing emotional support to patientsduring the treatment process [5]. In addition, nurses may be occupationally exposed to the drugs during drug preparation, intravenous administration, specialized stages in administration (e.g., intraperitoneal, pleural, pericardial, and cerebrospinal fluid), drug delivery, or waste disposal, as well as cleaningdrug leaks [8]. The significant amounts of antineoplastic drugs can lead tocontamination through eating, drinking,and inhaling airborne powders and particles, as well as hand-mouth contact,unprotected skin and mucous membranes, or needle sticking [9].Based on the results of hospital studies,thelevel of antineoplastic drugs is significant in the air, surfaces, gloves, and various parts of the treatment team [10].Organizations such as NIOSH and Oncology Nurses Society (ONS) have recommended safety guidelines for antineoplastic drugs.Some of the guidelines include modifying the physical structure of the workplace in terms of isolating the drug preparationspace, minimizing nurses’ contact with the drug, applying standard work methods, and utilizing personal protective equipment to achieve the minimum contact of antineoplastic drugs with the skin or respiratory system of nurses [11, 12].

Nurses should be aware of the risks and safe handling ofantineoplastic drugs toenhance the safety of both patients and themselves.The use of biological safety cabinets (BSCs) against inhalation exposure during drug preparation andnonpermeable gowns and double glovesareamong the methods recommended for reducingexposure to antineoplastic drugs.Other approaches includewearingtwo pairs of powder-free latex gloves,and face shields or goggles,protecting the facefrom drug splashes, as well as utilizing surveillance systems and precise procedures to reduce exposure opportunities.Theoccupational exposure to antineoplastic drugs can decreaseif all precautions are taken consistently [13].

Recent studies have shown thecontamination of the workplace and healthcare workers, especially nurses,and indeveloping countries,although the guidelines for the safe use of antineoplastic drugs have been in place for the past 20 years[14]. The resultshave raised serious concerns about nurses’knowledge and performance on safe procedures.The lack of knowledge, as well as failingtofollow the guidelinesareconsidered the mainreasons for the unsafe handlingof antineoplastic drugs.Accordingly, the guidelines cannot guarantee safe behaviors, and awareness is an important factor in changing individuals’ performance [15].

Given that an unsafe drug treatment process leads toADRs and sometimes irreversible disorders, standard drug treatment is required to reducetheside effects of exposure to antineoplastic drugs among patients and nurses and improve nurses’ performance in managing occupational exposure and safe medication.Thus,regarding the safety tips in using the drugs, the awarenessof oncology nursesis important. Therefore, the present study soughtto determinethe effects of standard guidelines education on the safe handling of antineoplastic drugs amongoncologynurses.

## Method

### Study design

This study with a quasi-experimental one-group pretest-posttest design was performed amongallnurses employed in theoncologywards of two educationalhospitals in Ardabil city during 2020. The ethical approvals were obtained from the Bioethics Committee of the Ardabil University of Medical Sciences, Iran (Approval No. IR.ARUMS.REC.1398.040). All participants gave written informed consent.

### Participants

The study population consisted of allthe nurses working in theoncologywards of Imam Khomeini and Bu-Ali educational hospitals in Ardabil(*n*=35) as the only centers for chemotherapy in the province.The inclusion criteria included full-time occupationin the mentioned wards, having at least three months of experience in the oncologywards, and agreeing to participate in this study. 32 nurses were eventuallyentered into the study.

In the present study,the researcher attendedthe hospital director and nursing offices of the intended hospitals, followed by introducingthem to the oncologywards.Additionally, she explained the research objectives, assured the information confidentiality, and proceeded to obtain informed written consent from the participants.Further,a questionnaire and a performance checklist were respectively utilized to examinethe nurses’ knowledge, as well asperformance atall stages of the drug treatment process.

After the initial evaluation, the nurses participated a two-day workshop.In theworkshop, an oncologist and an experienced nursing professor presented the content of the training package in the form of lectures, group discussions, film screenings, and educational bookletsto the individuals. The content of the training package was extracted from the latest guidelines for the management of antineoplastic drugs services (National Cancer Management Program (2017)) and standards for nursing services in chemotherapy.Finally, the nurses’ knowledge and performance were re-evaluatedthree months after the end of the training course.

### Data collection and statistical analysis

Two tools were applied for data collection.First,a researcher-made questionnaire to assess the nurses’ knowledge of the standard guidelines for handlingantineoplastic drugs and second,an observational checklist to examinetheperformance of the nurses by the standard guidelinesamong inpatient and outpatient wards.

The nurses’ knowledge questionnaire consisted of two parts (37 questions).The first part included the demographic characteristics of nurses such as age, sex, marital status,as well as thelevel of education, typeandduration of employment in the oncologyward, type of shift, and numberof working hours per week. Additionally information on the completion ofprevious training workshops, along with standard environment and facilities, periodic tests, and occupational accidents were also obtained.The second part of the questionnaire assessed theirawareness of the proper principles of drug preparation, ways of drug absorption,ADRs of antineoplastic drugs, correct method of using personal protective equipment, and knowledge of the necessary measures in case of occupational exposure to antineoplastic drugs and waste disposal(for example, exposure through inhalation and the use of N95 masks to protect against airborneantineoplasticparticles,, the equal effectiveness of disinfectant solution and soap and water to remove the remnants of antineoplastic drugs, managing accidental ocular exposure to antineoplastic drugs by washing the face, etc.). In the questionnaire, the score of each itemis either1 (yes) or 0 (I do not knowand no). Receiving a score of 1forall questionsrepresents the desired level of knowledge, while a score of 0 reflects a knowledge gap.

Thechecklistcontained four main subscales of drug preparation (before injection), injection, leakage,and waste disposal performance(18, 14, 4, and 6 items, respectively)based on the guidelines of the Ministry of antineoplastic Services Management(2017), as well asnursing services standards [16].Some items included the following: wash your hands with soap and water before preparing antineoplastic drugs, wear non-absorbable disposable gownswith long-sleeved goggles when injecting, usea face shield when preparing drugs,place the devices used in the injection of antineoplastic drugs in impermeable bags, etc.The score of each itemwas0 (no)or1(yes) so thatthe nurses’ performance scores in the safe drug treatment processcould varyfrom 0 to 42.Further, the scores of 0-8, 9-17, 18-25, 26-33, and 34-42 indicate a very poor, poor, medium, good, and excellent performance, respectively.

In order to validate the content of the training package and the above-mentioned tools, they were provided to 10 nursing professors and their expert opinions were received. Furthermore,content validity ratio coefficients (CVR) and content validity index (CVI) were used to measure the quantitative content validity index.Based on the Waltz and Basel method,CVR was calculatedat 93.2 for the knowledge questionnaire and at 92.8 performance checklist.Regarding the reliability, Cronbach’s alpha coefficient was computed0.74 and 0.72 for the questionnaireand checklist, respectively.

The data were analyzed using descriptive (mean and standard deviation) and inferential statistics (Pearson’s correlation coefficient and t-test) in SPSS 22 software.

## Results

### Participants’ demographics

In thisstudy, most nurses were female (96.9%) with a mean age of 32.68±4.70 years.The mean work experience in the oncologywardwas 4.31±3.53 years.(Table 1).

**Table 1:** Demographic characteristics of the studied nurses

Nurses’ characteristics	Mean ± SD
Age	32.68±4.70
Years of experience in chemotherapy drug administration	4.31±3.53
Working hours during the week	46.34±5.72

### Description and correlation of major variables

Based on the results related to the participants’knowledge of the standard guidelines for handlingantineoplasticdrugs, the mean and standard deviation of their knowledge scores was 59.56±6.41 before the intervention, which increasedto 66±4.82 three months after training(*P*=0.001) (Table 2). In the pre-intervention stage, 24 nurses (75%) possessed excellent knowledge, while 29 (90.7%) obtained excellent knowledge scoresfollowing the training workshop.

**Table 2:** Difference between mean scores of nurses’ knowledge regarding antineoplastic drugs safe handling pre andpost guidelines intervention

Knowledge	Mean ± SD	Min	Max	P-value
Pre-intervention	59.56±6.41	11	36	0.001*
Post-intervention	66±4.82	20	37	

*Paired Sample Test

As shown in Tables 3 and 4, the meanscores (±standard deviation) of drug preparation and injection time were 0.56±0.09 and 0.41±0.10 respectively which increased to 0.79±0.06, 0.78±0.07respectively after the training. Moreover, mean scores for leakage, waste disposal, and overall performance were initially0.07±0.02, 0.48±0.10, and 18.96±2.54 respectively. These scores were raised to 0.50±0.20, 0.76±0.09, and 32.03±2.45 (*P*<0.001) respectively.

**Table 3:** Difference between mean scores of nurses’ safe handling practices of antineoplastic drugs pre and postguidelines intervention

Practice	Mean ± SD	Min	Max	P-value
Pre-intervention	18.96±2.54	14	23	0.001*
Post-intervention	32.03±2.45	28	38	

*Paired Sample Test

During the pre-intervention stage, 30 nurses (93.7%) exhibited moderate performance and no oneperformedwell.However, 29 participants (90.7%) hada good performance and no poor performance was observed after training.

**Table 4:** Difference between mean scores of nurses’ safe handling practices of antineoplastic drugs in the indicators of drug preparation, drug injection, drug leakage, and waste disposal pre and postguidelines intervention

Practice Items	Pre-intervention	Post-intervention	T-test	P-value
	**Mean ± SD**	**Mean ± SD**		
Drug preparation	0.56±0.09	0.79±0.06	-12.20	0.001
Drug injection	0.41±0.10	0.78±0.07	-15.91	0.001
Drug Leakage	0.07±0.02	0.50±0.20	-13.04	0.001
Waste disposal	0.48±0.10	0.76±0.09	-10.65	0.001

## Discussion

Based on the results of the present study, the level of the nurses’knowledge onthe safe handlingof antineoplastic drugs statistically significantly increasedfollowingtrainingthe standard guidelines,Regarding the participants’ performance, a statistically significant difference was observedin the mean scores before and after the intervention. In addition, their performance significantly improvedfollowing training in all stages of drug preparation, drug administration, drug leakage, andwaste disposal. This clearly demonstrated the positive effect of the intervention onthe safe handling of antineoplasticdrugs among oncologynurses.An increase in the nurses’knowledge and performance can be attributed to the effectiveness of the trainingcoursesonenhancingtheir knowledge and performance about antineoplastic drugs.Further, theresultsreflected the nurses’ tendency to gain knowledge onantineoplastic drugs for protecting themselves and their patients from the ADRs of these drugs. Most nurses complained about the lack of guidelines for using antineoplastic drugs in the wardwhich were approved by the Ministry of Health and Medical Education, and written training programs on how to handle these drugs, as well as their prescription process.The educational pamphlet focused on standard guidelines for handlingantineoplastic drugs among nurses, which contained all the necessary informationonantineoplastic drugs, as well as improving the knowledge and practice among nurses.

The results of most studies in this field confirmed the effect of the training interventions on nurses’ knowledge and performance.For example, Hazrati et al. (2008) reported an improvementin the knowledge and performance of oncology nurses to a good and excellent level after training [16]. Additionally, TaghizadehKermani et al. (2015) and Mohsen and Fareed(2013) found a significant increase inthe knowledge and performance of oncology nursesfollowing a short training course, as well as implementing the training protocol, respectively [17, 18].Further, Shrestha et al. (2017) suggested thatatraining intervention positively affectsthe knowledge of nurses’ regarding the safe use of antineoplasticdrugs in Nepal [19].Mahdy et al. (2017) also conducted a study titled“the effect of safetyguidelines for antineoplastic drugs on the knowledge and practice of nurses in Ain al-Shams hospitals in Egypt”, in which the results were consistent with the findings of the present study [20].According toKoulountiet al.(2019), the nurses receiving specialized training in antineoplastic drugs represent higher performance compared to the others[21].These results were also confirmed by the studies ofBolbol et al. (2016), Karpagam et al. (2017), Keat et al. (2013), Jeong et al. (2015), Zayed et al. (2019), and Angel and Soli (2017)which showed that the implementation of safetyguidelines for antineoplastic drugs has a positive effect on improving the knowledge and performance of oncology nurses and nursing students [22–27].

In this study, all nurses of two main chemotherapy centers in Ardabil received the necessary training on how to handleantineoplastic drugs according to the latest guidelines of the Ministry of Health and Medical Education of Iran.In addition, an educational package was distributed among all oncology nurses.These interventions were found to improve the the health of nurses and to increase in the quality of nursing services in dealing with chemotherapy drugs.

## Limitations

This study has a number of limitations including its small number of samples and the lack of a control group. The reason for both issues is the small number of nurses working in Ardabil chemotherapy wards.

## Conclusions

All in all, the resultsindicate anincrease in the knowledge and performance of the oncologynurses on the safe handling ofantineoplastic drugs after training according to the standard guidelines.Thus, raising the knowledge and improving the performance of nurses’ in the field of the safe management of antineoplastic drugs is considered essential in the planning and policies of the healthcare system.

## Data Availability

The datasets used and/or analyzed during the current study areavailablefrom the corresponding author on reasonable request.

## Abbreviations

NIOSH: National Institute of Occupational Safety and Health
ONS: Oncology Nurses Society
OSHA: Occupational Safety and Health Administration
BSCs: Biological Safety Cabinets

## References

[1] Sung H, Ferlay J, Siegel RL, Laversanne M, Soerjomataram I, Jemal A, Bray F (2021). Global Cancer Statistics 2020: GLOBOCAN Estimates ofIncidence and Mortality Worldwide for 36 Cancers in 185Countries. CA. Cancer J Clin.

[2] Amirkhah R, Naderi-Meshkin H, Mirahmadi M, Allahyari A, Sharifi HR (2017). Cancer statistics in Iran: towards finding priority for prevention and treatment. Multidisciplinary Journal of Cancer Pathophysiology.

[3] Alehashem M, Baniasadi Sh (2018). Safe handling of anti-neoplastic drugs in the university hospitals: a descriptive survey study among oncology nurses. Int J Cancer Manag.

[4] Shahrasbi AA, Afshar M, Shokraneh F, Monji F, Noroozi M, Ebrahimi-Khojin M (2014). Risks to health professionals from hazardous drugs in Iran: a pilot study of understanding of healthcare team to occupational exposure to cytotoxics. EXCLI J.

[5] Nwagbo SE, Ilesanmi RE, Ohaeri BM, Oluwatosin AO (2017). Knowledge of chemotherapy and occupational safety measures among nurses in oncology units. J Clin Sci.

[6] Graeve CU, McGovern PM, Alexander B, Church T, Ryan A, Polovich M (2017). Occupational exposure to antineoplastic agents: an analysis of health care workers and their environments. Workplace Health Saf.

[7] Callahan A, Ames NJ, Manning ML, Touchton-Leonard K, Yang L, Wallen GR (2016). Factor influencing nurses’ use of hazardous drug safe-handling precautions. Oncol Nurs Forum.

[8] DeJoy DM, Smith TD, Woldu H, Dyal M-A, Steege AL, Boiano J (2017). Effects of organizational safety practices and perceived safety climate on PPE usage, engineering controls, and adverse events involving liquid antineoplastic drugs among nurses. J Occup Environ Hyg.

[9] Johnson T (2017). Long-term care: safe drug handling of oral chemotherapy. ASCP.

[10] Aristizabal-Pachon AF, Castillo WO (2019). Genotoxic evaluation of occupational exposure to antineoplastic drugs. Toxicol Res.

[11] Al-Azzam SI, Awawdeh BT, Alzoubi KH, Khader YS, Alkafajei AM (2015). Compliance with safe handling guidelines of antineoplastic drugs in Jordanian hospitals. J Oncol Pharm Pract.

[12] Bernabeu-Martinez MA, Merino MR, Gago JMS, Sabucedo LMA, Wanden-Berghe C, Sanz-Valero J (2018). Guidelines for safe handling of hazardous drugs: a systematic review. PLoS One.

[13] Connor TH, MacKenzie BA, DeBord DG, Trout DB, O’Callaghan JP. NIOSH list of antineoplastic and other hazardous drugs in healthcare settings 2016. Department of Health and Human Services. Centers for Disease Control and Prevention: National Institute for Occupational Safety and Health; 2016.

[14] Jung J, Park JY (2019). Factors influencing compliance with safety guidelines of anticancer drugs among nurses in general hospitals. Asian Oncol Nurs.

[15] Guideline (2017). preventive chemotherapy to control soil-transmitted helminth infections in at-risk population groups. WHO Library Cataloguing-in-Publication Data.

[16] Hazrati M, Raeisi H, Torabizadeh K, Pasyar N (2008). The effect of education standard instructions for working with anti-neoplasm drugs on the drug delivery process in chemotherapy wards affiliated to Shiraz University of Medical Sciences. Hormozgan Medical Journal.

[17] Taghizadeh Kermani A, Hosseini S, Salek R, Pourali L (2015). Improving knowledge and attitude of nurses working in chemotherapy wards through a short educational course: a successful experience in Mashhad. Future of Medical Education Journal.

[18] Mohsen MM, Fareed ME (2013). Chemotherapy safety protocol for oncology nurses: it’s the effect on their protective measures practices. WASET.

[19] Shrestha(Rai) DK, Lama S, Badu A, Mandal GN (2017). Impact of educational intervention on knowledge regarding safe handling of cytotoxic drugs among the nursing personnel working in BPKIHS. Health Renaissance.

[20] Mahdy NE, Abdel Rahman A, Hassan H (2017). Cytotoxic drugs safety guidelines: Its effect on awareness and safe handling practices of oncology nurses. IOSR- JNHS.

[21] Koulounti M, Roupa Z, Charalambous C, Noula M (2019). Assessment of nurse’s safe behavior towards chemotherapy management. Mater SocioMed.

[22] Bolbol SA, Hassan AA, El-Naggar SA, Zaitoun MF (2016). Role ofoccupational health and safety program in improving knowledge and practice among nurses exposed to chemotherapy at Zagazig university hospitals. Egypt J Occup Med.

[23] Karpagam K, Mangalagowr P, Aruna S (2017). Effectiveness of planned teaching program on safe handling of chemotherapy drugs among staff nurses. Int J Pharm Biol Sci.

[24] Keat CH, Sooaid NS, Yun CY, Sriraman M (2013). Improving safety-related knowledge, attitude and practices of nurses handling cytotoxic anticancer drug: pharmacists’ experience in a general hospital, Malaysia. Asian Pac J Cancer Prev.

[25] Jeong KW, Lee B-Y, Kwon MS, Jang J-H (2015). Safety management status among nurses handling anticancer drugs: nurse awareness and performance following safety regulations. Asian Pac J Cancer Prev.

[26] Zayed HA, Saied SM, El-Sallamy RM, Shehata WM (2019). Knowledge, attitudes and practices of safe handling of cytotoxic drugs among oncology nurses in Tanta university hospitals. Egyp J Occup.

[27] Angel RG, Soli TK (2017). Effectiveness of structured education on safe handling and disposal of chemotherapeutic drugs among nursing students. Int J Pharmacol Clin Res.

